# Supplementary material for: Electrochemical Sensor Based on Molecularly Imprinted Polymer for the Detection of Cefalexin
Source: Biosensors (Basel). 2019 Feb 27;9(1):31. doi: 10.3390/bios9010031 (PMC6468396; doi:10.3390/bios9010031)
Supplement: Supplementary file 1 [file biosensors-09-00031-s001.pdf]

Electrochemical sensor based on molecularly imprinted polymer for the detection of cefalexin

Bogdan Feier[a#], Adrian Blidar[a#], Alexandra Pusta[a], Paula Carciuc[a], Cecilia Cristea[a]*

[a]*Analytical Chemistry Department, Faculty of Pharmacy,*

Iuliu Haţieganu University of Medicine and Pharmacy, 4 Pasteur St., 400349 Cluj-Napoca, Romania

*ccristea@umfcluj.ro;

Phone: +40264597256 (extension: 2840); Fax: +40264597257

# equal contribution

Supplementary information

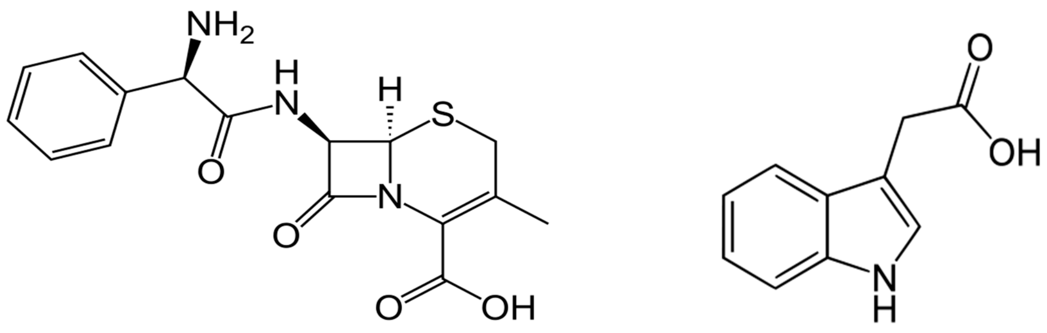

Figure S1. CFX (left) and I3AA (right) structures

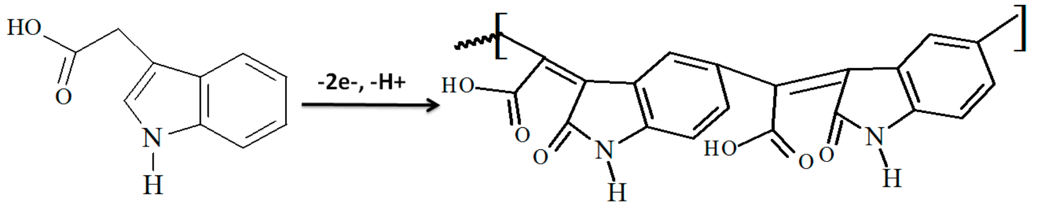

Figure S2. The polymerization mechanism

Table S1. Redox probe for the MIP-modified GCE

**Polymerization: 1 mM I3AA, 0.5 mM CFX, 5 cycles; Extraction: NaOH 0.1 M, 30 min; Incubation: 0.5 µM CFX, water, 30 min**						
**Redox probe**	**GCE**					
	**MIP**			**NIP**		
	S_{polym}	S_{extr}	S_{incub}	S_{polym}	S_{extr}	S_{incub}
$[Ru(NH_3)_6]^{3+}$	0.885	9.413	0.027	0.87	8.725	0.023
$[Fe(CN)_6]^{3-/4-}$	**0.995**	**6.320**	**0.436**	**0.995**	**1.138**	**0.176**
1,1'-ferrocenedimethanol	0.925	12.235	0.022	0.916	11.374	0.021

Table S2. Redox probe for the MIP-modified BDDE

**Polymerization: 1 mM I3AA, 0.5 mM CFX, 5 cycles; Extraction: NaOH 0.1 M, 30 min; Incubation: 0.5 µM CFX, water, 30 min**						
**Redox probe**	**BDDE**					
	**MIP**			**NIP**		
	S_{polym}	S_{extr}	S_{incub}	S_{polym}	S_{extr}	S_{incub}
$[Ru(NH_3)_6]^{3+}$	0.835	7.635	0.015	0.85	7.881	0.024
$[Fe(CN)_6]^{3-/4-}$	**0.986**	**8.348**	**0.532**	**0.993**	**0.735**	**0.123**
1,1'-ferrocenedimethanol	0.934	12.483	0.03	0.945	11.856	0.018

Table S3. Optimization of polymerization – DPV signal, on the GCE electrode

**Extraction: NaOH 0.1 M, 30 min; Incubation: 0.5 µM CFX, water, 30 min**							
**Parameter**	**Value**	**GCE**					
		**MIP**			**NIP**		
		S_{polym}	S_{extr}	S_{incub}	S_{polym}	S_{extr}	S_{incub}
**C_{mon} (mM) (C temp – 0.05 mM)**	0.1	0.907	5.210	0.311	0.957	2.425	0.284
	**1**	**0.932**	**4.870**	**0.358**	**0.978**	**2.310**	**0.292**
	5	0.981	3.920	0.243	0.99	1.485	0.287
**C_{temp} (mM) (C_{mon} – 1 mM)**	0.01	0.942	3.460	0.303	**0.978**	**2.310**	**0.292**
	0.05	0.932	4.870	0.358			
	0.1	0.921	6.650	0.398			
	**0.5**	**0.973**	**8.820**	**0.501**			
	1	0.872	9.750	0.373			
**No of cycles**	2	0.93	7.240	0.382	0.963	2.670	0.294
	**5**	**0.973**	**8.820**	**0.501**	**0.978**	**2.310**	**0.292**
	10	0.987	6.230	0.238	0.985	1.860	0.107

Table S4. Optimization of polymerization – DPV signal, on the BDDE electrode

Extraction: NaOH 0.1 M, 30 min; Incubation: 0.5 µM CFX, water, 30 min							
Parameter	Value	BDDE					
		MIP			NIP		
		S_{polym}	S_{extr}	S_{incub}	S_{polym}	S_{extr}	S_{incub}
C_{mon} (mM) (C temp – 0.05 mM)	0.1	0.915	6.930	0.327	0.948	1.432	0.271
	**1**	**0.927**	**6.340**	**0.398**	**0.965**	**1.235**	**0.257**
	5	0.972	5.280	0.289	0.987	0.845	0.238
C_{temp} (mM) (C_{mon} – 0.05 mM)	0.01	0.958	5.670	0.297	**0.965**	**1.235**	**0.257**
	0.05	0.927	6.340	0.398			
	0.1	0.949	7.860	0.475			
	**0.5**	**0.987**	**9.530**	**0.604**			
	1	0.898	10.230	0.412			
No of cycles	2	0.953	7.830	0.432	0.924	1.023	0.246
	**5**	**0.987**	**9.530**	**0.604**	**0.965**	**1.235**	**0.257**
	10	0.991	8.010	0.367	0.982	0.723	0.209

Table S5. Optimization of polymerization – EIS signal, on the GCE electrode

Extraction: NaOH 0.1 M, 30 min; Incubation: 0.5 µM CFX, water, 30 min							
Parameter	Value	GCE					
		MIP			NIP		
		S_{polym}	S_{extr}	S_{incub}	S_{polym}	S_{extr}	S_{incub}
C_{mon} (mM) (C temp – 0.05 mM)	0.1	10.763	0.839	0.467	22.267	0.708	0.397
	**1**	**13.701**	**0.827**	**0.558**	**45.167**	**0.698**	**0.413**
	5	52.34	0.794	0.325	97.023	0.598	0.403
C_{temp} (mM) (C_{mon} – 0.05 mM)	0.01	16.241	0.774	0.434	**45.167**	**0.698**	**0.413**
	0.05	13.701	0.827	0.558			
	0.1	11.94	0.867	0.672			
	**0.5**	**39.034**	**0.901**	**1.011**			
	1	9.835	0.908	0.597			
No of cycles	2	14.015	0.879	0.619	26.027	0.723	0.416
	**5**	**39.034**	**0.901**	**1.011**	**45.167**	**0.698**	**0.413**
	10	79.678	0.863	0.315	66.687	0.651	0.121

Table S6. Optimization of polymerization – EIS signal, on the BDDE electrode

Extraction: NaOH 0.1 M, 30 min; Incubation: 0.5 µM CFX, water, 30 min							
Parameter	Value	BDDE					
		MIP			NIP		
		S_{polym}	S_{extr}	S_{incub}	S_{polym}	S_{extr}	S_{incub}
C_{mon} (mM) (C temp – 0.05 mM)	0.1	10.775	0.874	0.487	18.345	0.589	0.372
	1	**12.743**	**0.865**	**0.662**	**28.572**	**0.563**	**0.347**
	5	34.716	0.842	0.407	75.94	0.46	0.314
C_{temp} (mM) (C_{mon} – 0.05 mM)	0.01	22.812	0.85	0.423	**28.572**	**0.563**	**0.347**
	0.05	12.743	0.865	0.662			
	0.1	18.609	0.888	0.905			
	**0.5**	**76.345**	**0.907**	**1.525**			
	1	8.813	0.911	0.7			
No of cycles	2	21.276	0.893	0.763	12.346	0.508	0.329
	**5**	**76.345**	**0.907**	**1.525**	**28.572**	**0.563**	**0.347**
	10	102.123	0.891	0.583	55.321	0.421	0.267

Table S5. Optimization of extraction – DPV signal

Polymerization: 1 mM I3AA, 0.5 mM CFX, 5 cycles; Extraction: NaOH 0.1 M, 30 min; Redox probes:10 mM Fe^{3+} – DPV, 10 mM $[Fe(CN)_6]^{-3/-4}$ -EIS									
**Solvent**	**Time (minutes)**	**GCE**				**BDDE**			
		**MIP**		**NIP**		**MIP**		**NIP**	
		S_{extr}	S_{incub}	S_{extr}	S_{incub}	S_{extr}	S_{incub}	S_{extr}	S_{incub}
**MeOH**	5	6.410	0.352	1.813	0.284	7.762	0.415	1.627	0.203
	15	7.560	0.391	2.840	0.312	8.853	0.521	2.154	0.225
	30	9.750	0.448	3.152	0.287	10.236	0.557	3.231	0.248
	60	11.380	0.37	3.750	0.279	12.053	0.456	3.689	0.235
**NaOH 0.1M**	5	4.510	0.345	1.423	0.289	5.387	0.423	0.926	0.25
	15	6.330	0.404	1.873	0.29	7.325	0.527	1.085	0.253
	**30**	**8.820**	**0.501**	**2.310**	**0.292**	**9.530**	**0.604**	**1.235**	**0.257**
	60	9.280	0.435	2.458	0.283	9.864	0.461	1.367	0.239
**PBS, pH 7.4**	5	2.320	0.345	0.856	0.285	3.213	0.423	0.763	0.244
	15	4.530	0.404	1.341	0.283	4.345	0.525	0.959	0.248
	30	6.210	0.467	1.557	0.278	5.847	0.585	1.183	0.245
	60	6.850	0.374	1.625	0.277	6.329	0.46	1.348	0.247

Table S6. Optimization of extraction – EIS signal

Polymerization: 1 mM I3AA, 0.5 mM CFX, 5 cycles; Extraction: NaOH 0.1 M, 30 min; Redox probe: 10 mM $[Fe(CN)_6]^{-3/-4}$									
**Solvent**	**Time (minutes)**	**GCE**				**BDDE**			
		**MIP**		**NIP**		**MIP**		**NIP**	
		S_{extr}	S_{incub}	S_{polym}	S_{extr}	S_{polym}	S_{polym}	S_{extr}	S_{polym}
**MeOH**	5	0.866	0.544	0.644	0.398	0.886	0.709	0.624	0.252
	15	0.885	0.647	0.741	0.456	0.899	1.091	0.683	0.29
	30	0.908	0.81	0.759	0.404	0.911	1.257	0.764	0.331
	60	0.92	0.588	0.792	0.387	0.925	0.839	0.787	0.306
**NaOH 0.1M**	5	0.818	0.527	0.587	0.407	0.843	0.732	0.481	0.333
	15	0.863	0.679	0.653	0.409	0.881	1.114	0.52	0.337
	**30**	**0.899**	**1.011**	**0.697**	**0.414**	**0.905**	**1.525**	**0.553**	**0.348**
	60	0.903	0.771	0.71	0.395	0.908	0.854	0.577	0.314
**PBS, pH 7.4**	5	0.696	0.527	0.461	0.399	0.763	0.732	0.433	0.323
	15	0.821	0.677	0.572	0.395	0.813	1.104	0.489	0.331
	30	0.863	0.876	0.609	0.385	0.854	1.408	0.542	0.321
	60	0.873	0.597	0.619	0.384	0.867	0.849	0.574	0.328

Table S7. Optimization of incubation (DPV, EIS)

Polymerization: 1 mM I3AA, 0.5 mM CFX, 5 cycles; Extraction: NaOH 0.1 M, 30 min; Redox probes:10 mM Fe^{3+} – DPV, 10 mM $[Fe(CN)_6]^{-3/-4}$ - EIS									
**Solvent**	**Time (minutes)**	**GCE**				**BDDE**			
		**MIP**		**NIP**		**MIP**		**NIP**	
		**DPV**	**EIS**	**DPV**	**EIS**	**DPV**	**EIS**	**DPV**	**EIS**
**Water**	5	0.369	0.601	0.205	0.253	0.435	0.772	0.179	0.221
	15	0.430	0.777	0.237	0.306	0.546	1.203	0.213	0.268
	**30**	**0.501**	**1.020**	**0.292**	**0.412**	**0.604**	**1.560**	**0.257**	**0.347**
	60	0.505	1.023	0.298	0.420	0.609	1.540	0.261	0.351
**PBS, pH 7.4**	5	0.372	0.610	0.204	0.261	0.440	0.792	0.205	0.258
	15	0.435	0.792	0.276	0.381	0.553	1.237	0.231	0.300
	30	0.506	1.035	0.293	0.414	0.612	1.567	0.262	0.352
	60	0.508	1.041	0.301	0.433	0.615	1.624	0.265	0.357

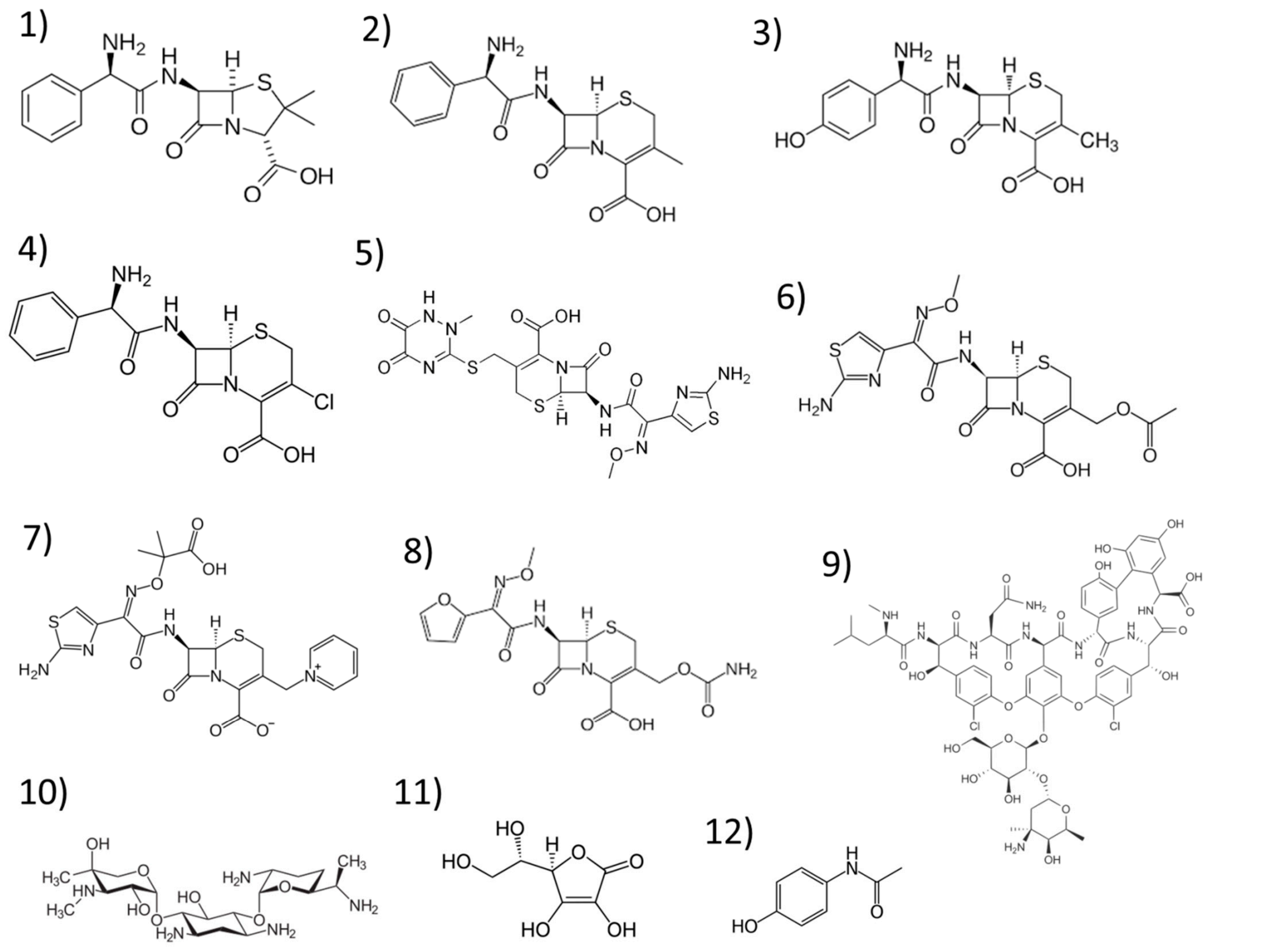

Figure S3. The structures of the molecules for which the MIP sensor was tested: 1) Ampicillin (AMP), 2) Cefalexin (CFX), 3) Cefadroxil (CFD), 4) Cefaclor (CFC), 5) Ceftriaxone (CFTXO), 6) Cefotaxime (CFTX), 7) Ceftazidime (CFTZ), 8) Cefuroxime (CFOX)), 9) Vancomycin (VAN), (10) Gentamicin (GEN), 11) Ascorbic acid (AA) and 12) Acetaminophen (APAP)

Table S8. Comparative analytical performance of different methods for CFX detection

No.	Method of detection	Linear range (ng/mL)	LOD (ng/mL)	Interferences	Real-life samples	Observations	Ref.
1.	Visualized microarray	8.20–68.60	8.20	Melamine, aflatoxin	Milk	Complex work protocol<br>Requires use of monoclonal antibodies (expensive, liable)	(1)
2.	MIP for SPE+ HPLC			Ceftazidime, ampicillin and tetracycline hydrochloride	Milk	Complex work protocol<br>Requires qualified staff<br>Interferences on C18 SPE column due to non-specific interactions	(2)
3.	MISPE-UHPLC–MS/MS		2.24	Cefthiofur, cefazolin, cefquinome, cephapirin, cephalexin and cephalonium fluoroquinolones, doxycyciline	Milk	Toxic reagents (ACN, MeOH)<br>Complex work protocol<br>Requires qualified staff<br>Low recovery rates for cephalexine<br>Similar recovery rates between analytes and interferences	(3)
4.	Spectrophotometric UV	800.00–2800.00	168.00	Starch, lactose, glucose, sucrose, and gum acacia	Pharm. form.	Requires derivatisation of the probe with 1,2-naftoquinone-4-sulphonic	(4)
5.	DPV on bare BDDE	173.80-243313.00	34.74	acetaminophen, ascorbic acid, glucose, other cephalosporins	River water, pharm. form., human urine	DPV peak for cephalexin decreases in the presence of other cephalosporins	(5)
6.	SWV on heated glassy carbon electrode	208.55-17379.50	52.00	Na+ , K+ , Ca2+, Cu2+, Mg2+, Cu2+, Pb2+, Zn2+, CO3 2−, SO4 2−, NO3 − , Cl− , dextrin, amylum glucose, 100 NH4 + , Fe2+, SO3 2−, lactose, glucose and sucrose, and citric acid, urea and uric acid	Pharm. form.	Requires alkaline hydrolysis of the analyte before the method can be applied	(6)

7.	HPLC with UV detection	25.00 to 1600.00	10.00	-	Bovine milk	Harmful reagents (acetonitrile, glutaraldehyde)<br>Requires a protein exclusion step, using two columns and a column switching system	(7)
8.	LC–ESI–MS/MS	2.00–100.00	0.50	Simultaneous detection with Cefminox, cefotaxime, cefetametplvoxll, ceftazidime, cephalonium cefixime cefadroxil, cefazolin, cefuroxime, cefalotin, cefradine, cefapirin cefaclor cefonicid, cefamandole, cefepime, ceftriaxone, cefpirome, cefoperazone, ceftiofur, cefquinome	Pork muscle	Harmful reagents<br>Qualified staff	(8)
9.	Microbiological system	-	128.00	-	Ovine milk	Lacks selectivity<br>Requires Geobacillus stearothermophilus spores, which can contaminate the work environment	(9)
10.	Electrochemical oxidation by CV using the boron-doped diamond thin-film electrode	In the range of mM	-	-	-	Requires microwave plasma-assisted chemical vapor deposition of film<br>Deposition lasts about 10 h	(10)
11	Our MIP-based sensor	3.47 - 347.38	1.11 (BDDE) 1.70 (GCE)	Cefadroxil, cefaclor, ceftriaxone, cefotaxime, ceftazidime, cefuroxime, ampicillin, gentamicin, vancomycin, ascorbic acid, acetaminophen	Pharm. form., river water	-	This work

Pharm. form - pharmaceutical formulations; LOD –Limit of detection

References

1. Li Z, Li Z, Jiang J, Xu D. Simultaneous detection of various contaminants in milk based on visualized microarray. Food Control [Internet]. Elsevier; 2017 Mar 1 [cited 2018 Nov 1];73:994–1001. Available from:

https://www.sciencedirect.com/science/article/pii/S0956713516305539
2. Lata K, Sharma R, Naik L, Rajput YS, Mann B. Synthesis and application of cephalexin imprinted polymer for solid phase extraction in milk. Food Chem [Internet]. Elsevier; 2015 Oct 1 [cited 2018 Nov 1];184:176–82. Available from: https://www.sciencedirect.com/science/article/pii/S0308814615004732
3. Baeza AN, Urraca JL, Chamorro R, Orellana G, Castellari M, Moreno-Bondi MC. Multiresidue analysis of cephalosporin antibiotics in bovine milk based on molecularly imprinted polymer extraction followed by liquid chromatography-tandem mass spectrometry. J Chromatogr A [Internet]. Elsevier; 2016 Nov 25 [cited 2018 Nov 1];1474:121–9. Available from: https://www.sciencedirect.com/science/article/pii/S0021967316314546
4. Ali Ahmed SM, Elbashir AA, Aboul-Enein HY. New spectrophotometric method for determination of cephalosporins in pharmaceutical formulations. Arab J Chem [Internet]. Elsevier; 2015 Mar 1 [cited 2018 Nov 1];8(2):233–9. Available from: https://www.sciencedirect.com/science/article/pii/S1878535211002255
5. Feier B, Gui A, Cristea C, Săndulescu R. Electrochemical determination of cephalosporins using a bare boron-doped diamond electrode. Anal Chim Acta [Internet]. Elsevier; 2017 Jul 11 [cited 2018 Nov 1];976:25–34. Available from: https://www.sciencedirect.com/science/article/pii/S0003267017305597
6. Chen Y, Huang L, Lin Q. Rapid hydrolysis and electrochemical detection of cephalexin at a heated glassy carbon electrode. Int J Electrochem Sci. 2012;7(9):7948–59.
7. Oliveira R V., De Pietro AC, Cass QB. Quantification of cephalexin as residue levels in bovine milk by high-performance liquid chromatography with on-line sample cleanup. Talanta [Internet]. Elsevier; 2007 Feb 28 [cited 2018 Nov 1];71(3):1233–8. Available from: https://www.sciencedirect.com/science/article/pii/S0039914006004589
8. Li W, Shen H, Hong Y, Zhang Y, Yuan F, Zhang F. Simultaneous determination of 22 cephalosporins drug residues in pork muscle using liquid chromatography–tandem mass spectrometry. J Chromatogr B [Internet]. Elsevier; 2016 Jun 1 [cited 2018 Nov 1];1022:298–307. Available from: https://www.sciencedirect.com/science/article/pii/S1570023216302458
9. Nagel OG, Beltrán MC, Molina MP, Althaus RL. Novel microbiological system for antibiotic detection in ovine milk. Small Rumin Res [Internet]. Elsevier; 2012 Jan 1 [cited 2018 Nov 1];102(1):26–31. Available from: https://www.sciencedirect.com/science/article/pii/S0921448811004573
10. Chailapakul O, Aksharanandana P, Frelink T, Einaga Y, Fujishima A. The electrooxidation of sulfur-containing compounds at boron-doped diamond electrode. Sensors Actuators B Chem [Internet]. Elsevier; 2001 Dec 1 [cited 2018 Nov 1];80(3):193–201. Available from: https://www.sciencedirect.com/science/article/pii/S0925400501009121
